# Neural bases of social feedback processing and self–other distinction in late childhood: The role of attachment and age

**DOI:** 10.3758/s13415-020-00781-w

**Published:** 2020-03-05

**Authors:** Jonas G. Miller, Sharon Shrestha, Allan L. Reiss, Pascal Vrtička

**Affiliations:** 1 Center for Interdisciplinary Brain Sciences Research, Department of Psychiatry and Behavioral Sciences, Stanford University, Stanford, CA USA; 2 Departments of Radiology and Pediatrics, Stanford University, Stanford, CA USA; 3 Centre for Brain Sciences, Department of Psychology, University of Essex, Wivenhoe Park, CO4 3SQ UK; 4 Research Group “Social Stress and Family Health”, Max Planck Institute for Human Cognitive and Brain Sciences, PO BOX 500 355, 04303 Leipzig, Germany

**Keywords:** Social cognitive affective neuroscience, Late childhood, fMRI, Attachment, Age

## Abstract

Attachment plays a key role in how children process information about the self and others. Here, we examined the neural bases of interindividual differences in attachment in late childhood and tested whether social cognition-related neural activity varies as function of age. In a small sample of 8-year-old to 12-year-old children (*n* = 21/19), we used functional magnetic resonance imaging to measure neural responses during social feedback processing and self–other distinction. Attachment was assessed using child self-report. The social feedback processing task presented smiling and angry faces either confirming or disconfirming written information about participant performance on a perceptual game. In addition to observing main effects of facial emotion and performance, an increase in age was related to a shift from negative (i.e., angry faces/bad performance) to positive (i.e., smiling faces/good performance) information processing in the left amygdala/hippocampus, bilateral fusiform face area, bilateral anterior temporal pole (ATP), and left anterior insula. There were no effects of attachment on social feedback processing. The self–other distinction task presented digital morphs between children’s own faces and faces of their mother or stranger females. We observed differential activation in face processing and mentalizing regions in response to self and mother faces versus morphed faces. Furthermore, left ATP activity was associated with attachment anxiety such that greater attachment anxiety was related to a shift from heightened processing of self and mother faces to morphed faces. There were no effects of age on self–other distinction. We discuss our preliminary findings in the context of attachment theory and previous work on social evaluation and self–other processing.

**Electronic supplementary material:**

The online version of this article (10.3758/s13415-020-00781-w) contains supplementary material, which is available to authorized users.

The parent–child relationship provides the foundation for children’s learning about the self and others. The quality of this relationship is reflected in children’s attachment orientation to their parents, which, in turn, shapes how children respond to social situations (Bowlby, 1982). More specifically, attachment theory posits that sensitive versus insensitive caregiving experiences contribute to the development of different attachment-derived internal working models (IWMs)—cognitive schemas about the self, others, and social relationships in general—that have extended implications for social and emotional development (Bretherton & Munholland, 2008). In line with this perspective, a more secure attachment emerges from sensitive caregiving experiences and leads to an IWM that represents others as safe and dependable, and the self as worthy and capable of eliciting care when needed. Secure IWMs have consistently been associated with children’s positive development, including greater social competence and emotion understanding (Cooke, Stuart-Parrigon, Movahed-Abtahi, Koehn, & Kerns, 2016; Groh, Fearon, van Ijzendoorn, Bakermans-Kranenburg, & Roisman, 2017), higher quality peer relationships (Pallini, Baoiocco, Schneider, Madigan, & Atkinson, 2014), and fewer externalizing behaviors (e.g., aggression, conduct problems; Groh et al., 2017). Conversely, children who experience inconsistent and unpredictable caregiving are more likely to develop an insecure anxious attachment characterized by hypervigilance to signs of threat, whereas children whose caregivers are typically unavailable or unresponsive are likely to develop an insecure avoidant attachment characterized by a strong desire for independence and self-sufficiency. In terms of underlying attachment-derived IWMs, attachment anxiety and avoidance are generally associated with hyperactivation and deactivation, respectively, of an attachment system that orchestrates attachment-related needs and behaviors (Mikulincer & Shaver, 2007; Shaver & Mikulincer, 2002). Although these insecure IWMs represent meaningful adjustments to the environment in which children grow up, and thus are not inherently maladaptive, they have been associated with lower social competence and increased risk for psychopathology (Fonagy & Luyten, 2009; Groh et al., 2017). Social competence refers to the ability and skills that are important for engaging in positive social interactions, including effectively communicating feelings and emotion regulation. Early social competence is an important protective factor against the development of emotional and behavioral difficulties (Burt & Roisman, 2010).

Attachment-based interindividual differences in how children process information about the self and others can be investigated through studies of brain structure and function. Some studies have adopted a social neuroscience approach to test links between attachment and the neural correlates of social processing, mainly by using functional magnetic resonance imaging (fMRI; for recent reviews, see Letourneau, Hart, & MacMaster, 2017; Long, Verbeke, Ein-Dor, & Vrtička, 2020; Ran & Zhang, 2018; Swain et al., 2014; Vrtička, 2017; Vrtička & Vuilleumier, 2012). The vast majority of this work, however, has focused on adults, whereas only a handful of neuroimaging studies of attachment that focus on adolescents and children are available (Choi, Taylor, Hong, Kim, & Yi, 2018; Debbané et al., 2017; Leblanc, Degelih, Daneault, Beauchamp, & Bernier, 2017; Takiguchi et al., 2015; Vrtička, et al., 2014). Thus, we examined the potential links between attachment and the neural correlates of two aspects of social information processing—social feedback and self–other representation—with a special focus on late childhood.

In this study, children performed a task that included the processing of social feedback in the form of supportive or disapproving responses from others (Vrtička et al., 2008; Vrtička et al., 2014). Social feedback is often communicated by others’ emotional facial expressions, and children must integrate this feedback with information reflecting their objective performance on a corresponding task. Accordingly, these two different sources of social versus personal information can be congruent (e.g., other’s positive emotional expression combined with one’s own successful performance) or incongruent (e.g., other’s negative emotional expression combined with one’s own successful performance). Children may vary in their experiences of congruent versus incongruent, as well as positive versus negative, social feedback as a function of previous attachment-related experiences. For example, incongruent feedback could be one kind of inconsistent or unpredictable caregiving experience that is more common for children at risk for developing an anxious attachment orientation. Conversely, one might expect avoidantly attached children to have had less overall experience with caregiver-related social feedback integration due to the caregiver typically being unavailable or unresponsive. Attachment-related social feedback processing is likely important for social competence, as integrating different sources of social versus personal information may inform how children interact with others.

Attachment (in)security has been found to be related to neural sensitivity to different types of social feedback. Two previous studies of attachment have used the social feedback processing task in samples of young adults and adolescents (Vrtička et al., 2008; Vrtička et al., 2014). In the study of young adults (Vrtička et al., 2008), it was observed that securely attached participants showed increased ventral striatum activation in response to congruent positive feedback (i.e., social approval) and decreased amygdala activation in response to congruent negative feedback (i.e., perceived reproach). Diverging from this pattern, increased scores on attachment avoidance were associated with blunted ventral striatum and ventral tegmental area activation in response to congruent positive feedback, whereas increased scores on attachment anxiety were linked to heightened amygdala activation to congruent negative feedback. Collectively, these findings suggest that increased reward-related activation to social approval, and decreased threat-related activation to social reproach, may be neural features of IWMs found in securely attached adults. Furthermore, these findings indicate that attachment system deactivation related to attachment avoidance may negatively affect brain activity (i.e., down regulation) related to social reward and approval, whereas attachment system hyperactivation related to attachment anxiety may positively affect activity (i.e., hyperactivation) related to social threat and reproach. The study of adolescents obtained different findings, as anxious and avoidant attachment were differentially linked to neural sensitivity to congruency versus incongruency more generally. Although age and increased scores on anxious attachment were associated with heightened prefrontal and anterior insula activation in response to incongruent (versus congruent) feedback, increased scores on avoidant attachment were associated with heightened activation across a number of social-emotional information-processing brain regions (e.g., amygdala, hippocampus, anterior cingulate) in response to congruent (versus incongruent) feedback. Because of differences in methodological as well as procedural aspects (e.g., different magnetic field strengths, varying age-appropriate task descriptions) of the abovementioned two studies (also in comparison to the present investigation), the respective data cannot be directly integrated, unfortunately. The diverging patterns of activation in relation to attachment security, avoidance, and anxiety in the adult versus adolescent samples, however, may imply the presence of a developmental component to the establishment of attachment-derived IWMs. This being said, the neural bases of social feedback processing in childhood, and how these neural bases may be associated with attachment, are open questions.

Besides potentially guiding social feedback processing, attachment-derived IWMs could also affect other kinds of neural processes related to social competence, particularly mental representations of the self and others. Greater overlap in self–other representations, defined as inclusion of the characteristics of others into one’s self-concept, may occur more frequently in close compared to nonclose relationships (Aron & Fraley, 1999). Some research indicates that the degree of self–other overlap has implications for a number of attachment-related processes, including empathy for close others and the fostering of social bonds (Beckes, Coan, & Hasselmo, 2013; Cheng, Chen, Lin, Chou, & Decety, 2010; Galinsky, Ku, & Wang, 2005)—both of which are important aspects of social competence. Furthermore, it was shown that attachment insecurities may affect the representation of self–other similarity, with attachment anxiety leading to overestimation, but attachment avoidance to underestimation of self–other similarity (Mikulincer, Orbach, & Iavnieli, 1998). However, social neuroscience research on the putative neural substrates of self- and other-representation in association with attachment-derived IWMs has been rare. Debbané et al. (2017) used fMRI in adolescents ages 12 to 19 years while they attributed positive and negative adjectives to themselves and a close other (best same-sex friend) and found an association between increased scores on attachment anxiety and activity during both self- and other-representation in several brain areas related to emotion perception and regulation, mentalizing, and memory. Yet no study has directly considered attachment in relation to neural substrates of overlapping self-representations and other-representations in children.

Another experimental task that has been used in neuroimaging research to investigate self- and other-representation is based on self–other recognition or distinguishing between images of one’s own face and the face of another person (for a review, see van Veluw & Chance, 2014). More specifically, one can employ an experimental design where different faces are digitally morphed into each other (Harris, Young, & Andrews, 2012; Natu et al., 2016; Uddin et al., 2008). We aimed to build on this research by implementing a specific version of the self–other recognition task by morphing the child participant’s face into the face of his or her mother (versus an unknown female adult) to assess attachment-based differences in the neural correlates of self–other recognition.

Taken together, the current study was designed to improve our understanding of how attachment-derived IWMs in late childhood are represented at the level of brain functioning. To this end, we used fMRI to assess neural responses to two common social cognitive tasks designed to assess social feedback processing and self–other recognition. In addition to our main analyses, we were interested in exploring whether age would be associated with neural responses to these social cognitive tasks. A previous fMRI study of social feedback processing in 12-year-old to 19-year-old adolescents found evidence for age effects (Vrtička et al., 2014), potentially pointing to developmental differences. Thus, we conducted secondary, exploratory analyses to test whether age was associated with neural activity during two social cognitive tasks in our sample of 8-year-old to 12-year-old children. Given the limited number of previous studies, particularly with children, it was difficult to derive specific a priori hypotheses. We therefore used an exploratory whole-brain analytic approach for each task (with adequate correction for multiple comparisons) to determine which brain regions would be associated with attachment anxiety and avoidance, as well as with age.

## Method

### Participants

A total of 32 children (8–12 years of age) were recruited for this study from the local community through advertisements. Three children were adopted and were therefore removed from further analyses; this was done to prevent potential differences in attachment patterns related to preadoption caregiving that could not be controlled for and that may have influenced attachment formation with foster parents (Carlson, Hostinar, Mliner, & Gunnar, 2014). The remaining 29 children were living with their biological parents, except for two cases: one child was living with his or her biological mother and another same-sex parent, and one child was living with his or her mother, who carried the child to term, but conceived through a donor egg. These children were included in our analyses, given that in both cases, both parents were involved in childcare from birth. One child could not be scanned in the MRI machine due to strong anxiety. Thus, the current analyses included 28 children with available fMRI data.

Directly prior to scanning, all children underwent a mock-scanning procedure in an adjacent room at the Stanford Center for Cognitive and Neurobiological Imaging (CNI). The mock-scanning setup consisted of a wooden replica of an fMRI machine in which children were placed with earphones so that they could familiarize themselves with the different sounds to be heard during real fMRI scanning. In addition, a motion-tracking device was placed on children’s foreheads so that their head motion could be displayed on a visual target screen in real time. This procedure illustrated how much head motion would be acceptable during the subsequent real fMRI session. The mock scanning procedure lasted approximately 15-20 minutes.

Despite mock-scanning, for the social feedback processing task, five children had to be removed from analyses due to excessive head motion during fMRI (see fMRI Data Analysis section for details). Furthermore, two children demonstrated poor behavioral performance, defined as less than 50% “winning” trials (39.84% and 42.19%, respectively), despite the automatic performance-adjusting algorithm (see Experimental Tasks section for details). These participants were also removed from any further analyses. Thus, the final sample for the social feedback processing task analyses included 21 children (14 females, seven males, *M*_age_ = 10.43 years, *SD* = 1.18 years, age range: 8.22–12.81 years).

The face-morph task consisted of two conditions—morphing between mother–child faces and between stranger-female–child faces. For the mother–child block, two children were excluded from analyses due to not completing the entire scan and thus providing incomplete data. In addition, four children were excluded from analyses due to excessive head motion (see fMRI Data Analysis section for details). Thus, 22 children provided useable fMRI data for the mother–child block of the face-morph task (15 females, seven males, *M*_age_ = 10.49 years, *SD* = 1.16 years, age range: 8.22–12.81 years). In the stranger-female–child block, four participants were excluded due to incomplete data. In addition, five children were excluded due to excessive head motion (see fMRI Data Analysis section for details). Thus, the final sample for the face-morph stranger-female–child task included 19 children (13 females, six males, *M*_age_ = 10.69 years, *SD* = 1.00 years, age range: 9.19–12.81 years).

The fMRI scanning always started with the social feedback processing task and was followed by the face-morph task. For the second task, the mother–child and stranger-female–child blocks were counterbalanced.

### Attachment questionnaire

Children’s attachment orientation was assessed with an adaptation of the Experiences in Close Relationships Scale–Revised (ECR-R) for use with children and adolescents (ECR-RC; Brenning, Soenens, Braet, & Bosmans, 2011). As in the adult version of the questionnaire (Fraley, Waller, & Brennan, 2000), the ECR-RC comprises 36 items that assess avoidant (e.g. “It’s not easy for me to tell my mother a lot about myself”) and anxious (e.g. “I’m worried that my mother doesn’t really love me”) attachment dimensions (18 items for each dimension), with the items reformulated for better understanding by younger participants. The 36 items were rated on a 7-point scale ranging from 1 (*strongly disagree*) to 7 (*strongly agree*), with a neutral midpoint (4 = *agree/disagree*), and averaged for each attachment dimension. The ERC-RC was collected separately for reference to the mother and the father (the other same-sex parent in one case). To obtain more global measures of the children’s anxious and avoidant attachment to be used for both experimental tasks, ECR-RC values for mother and father were mean aggregated.

### Experimental tasks

The present study comprised two experimental tasks the children were asked to complete during fMRI scanning.

#### Social feedback processing

**Fig. 1:**
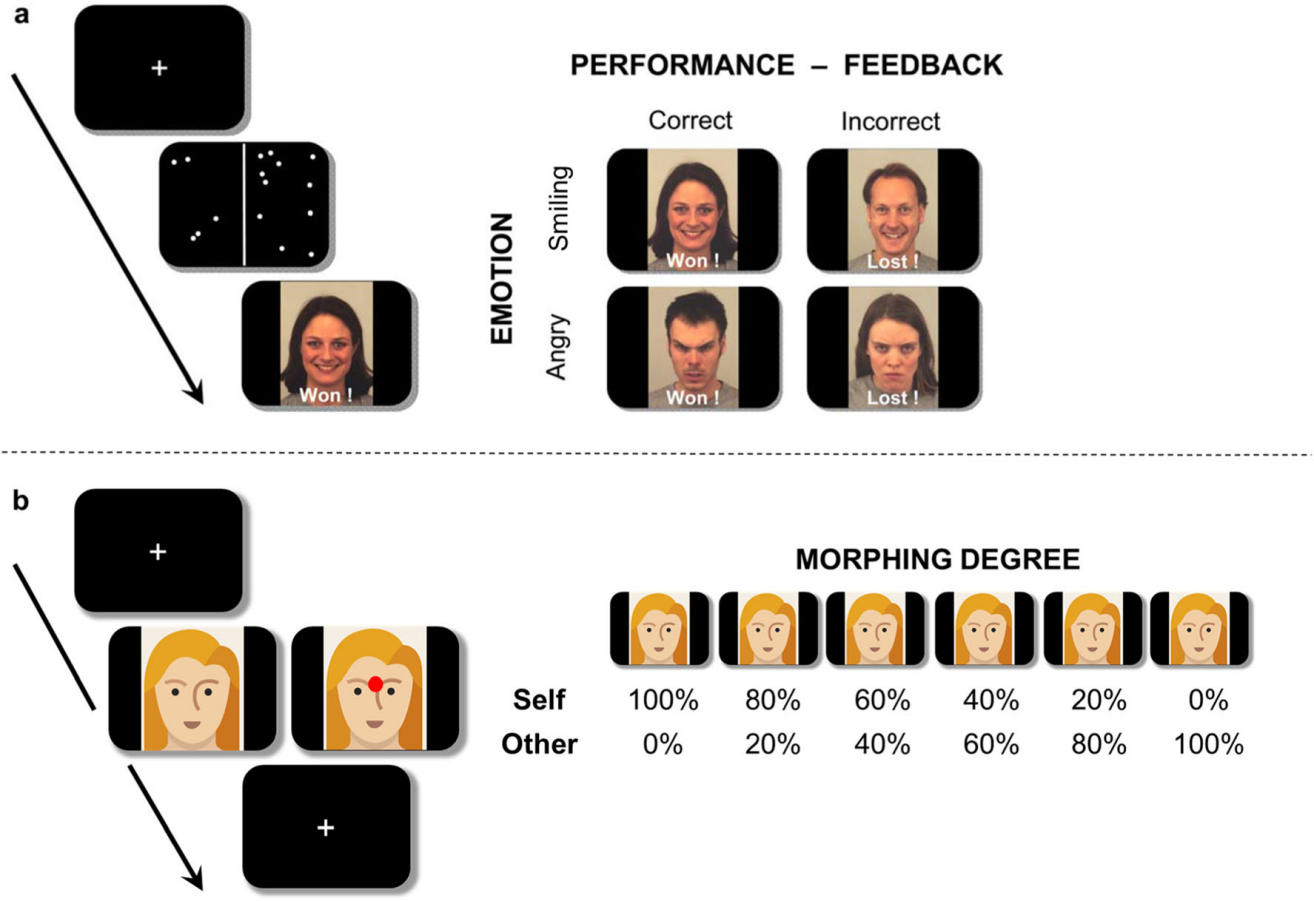
Illustration of the two experimental tasks. **a** Social feedback processing task, composed of a perceptual game consisting of trials that required rapidly counting and comparing the number of dots appearing in two dot clouds to the left and right of a centered line, and a visual social feedback screen after each trial (left panel). The social feedback was made up of a written performance feedback (either “Won” or “Lost”) and an emotional facial expression (either smiling or angry; right panel). **b** Face morph task, during which the own face, the mother’s face, or a stranger female face were shown to children (in 50% of cases with a red dot added between the eyes right above the nose; left panel) morphed into each other to 6 different degrees (right panel). Real photos cannot be shown due to data protection considerations. For more information, please refer to the text

In the social feedback processing task (Vrtička et al., 2008; Vrtička et al., 2014), children played a perceptual game consisting of trials that required rapidly counting and comparing the number of dots appearing in two dot clouds to the left and right of a centered line (see Fig. 1a, left panel). Task responses were given by participants using an MRI-compatible response pad. Feedback was provided after each trial, consisting of a word (“Won” or “Lost”) and an adult face (with a smiling or an angry emotional expression). The word always indicated the children’s objective performance during the preceding trial—correct responses were consistently paired with the word “Won,” and incorrect responses with the word “Lost.” The emotional facial expression, however, was not tied to objective performance, as smiling and angry expressions could be paired with both “Won” and “Lost” words. Thus, there were two congruent word–face pairings indicating social support (smiling face on won trials; SF-W) or social punishment (angry face on lost trials; AF-L), and two incongruent word–face pairings indicating schadenfreude/gloating (smiling face on lost trials; SF-L) and resentment (angry face on won trials; AF-W). This manipulation was designed to induce the perception of “friends” (congruent feedback) versus “foes” (incongruent feedback), as a smiling expression perceived after a successful trial (“Won”) has a very different social implication than a smiling expression seen after an unsuccessful trial (“Lost”). The task therefore delineates four well-differentiated and predefined congruent versus incongruent social evaluations of one’s own objectively evaluated performance (Vrtička et al., 2008; Vrtička et al., 2014; see Fig. 1a, right panel).

The total number of dots and the difference between the two display sides were adjusted online based on the children’s performance on preceding trials by either reducing the difference after each correct trial (minimum one dot) or increasing the difference after each incorrect trial (maximum five dots). This allowed us to maintain performance close to threshold and to obtain approximately equal numbers of correct and incorrect trials. In addition, to further ensure this equal distribution of correct and incorrect trials, occasional displays with 15 dots on both sides were inserted whenever performance exceeded 60% correct of two consecutive trials. None of the participants noticed these “trick” trials (Vrtička et al., 2008; Vrtička et al., 2014).

Children were informed that they would be presented with a difficult visual task in which the goal was to give as many correct responses as possible as fast as possible, and that we would measure their performance. Children were informed that each trial would be followed by feedback consisting of a word and a face. The word would provide feedback about their performance, and the face would provide emotional evaluations from people either being “friendly” or “unfriendly” (Vrtička et al., 2008; Vrtička et al., 2014).

The exact experimental setup was as follows. Each trial began with a jittered white central fixation cross on a black screen (for 3 to 7 s; average 3.5 s), followed by a brief visual display divided in two parts with a variable number of white dots on each side of the screen (presented for 500 ms). The dot display was followed by a black screen with a variable interval (jitter of 1,000 to 1,400 ms; average 1,200 ms), during which participants gave their response; and then by the visual feedback screen (1,500 ms). Face stimuli were color photographs of 16 different individuals (eight males) from the Karolinska Directed Emotional Faces set (KDEF; Lundqvist, Flykt, & Ohman, 1998). Each face identity was assigned to one condition only (two males and two females in each of the four feedback types, counterbalanced across participants). Thus, for a given child, a given face was always seen with the same expression (either smiling or angry) and the same feedback message (either positive “Won,” or negative “Lost”) throughout the task. Each face identity was repeated eight times in the corresponding conditions, in random order, resulting in 128 trials in total per child (total duration of approximately 15 min).

#### Face morph

The face-morph task consisted of pictures of the child’s own face (self), their mother’s face, and age-matched unknown female faces taken from an internal database at the Center for Interdisciplinary Brain Sciences Research (CIBSR; images of current and former adult female members of CIBSR). Child, mother, and stranger female pictures were taken in front of a standardized background with all hair removed from the face, no jewelry, no makeup, and with a neutral facial expression (mouth closed). All pictures were edited to appear in black and white and to only contain the face region. Children were shown their own faces (100% self condition), faces that were morphed between their own and another (mother or stranger female) to four varying degrees (80% self/20% other; 60% self/40% other; 40% self/60% other; 20% self/80% other), and faces of their mother or a stranger female (0% self). Digital morphs between children’s own and other faces were created using FantaMorph (Abrosoft; www.fantamorph.com). To ensure attention towards the faces, a red dot between the eyes directly above the nose was added to half of the faces, and children were asked to indicate by button press on an MRI-compatible response pad whenever a dot was present.

The task consisted of one block of trials containing the child’s self and their mother’s pictures (mother–child block) and one block of trials containing self and the age-matched unknown female’s pictures (stranger-female–child block). For each morphing degree, 20 repetitions were shown (10 of which included a red dot), resulting in a total of 120 trials per block with an approximate duration of 10 minutes. The exact experimental setup was as follows. After an initial instruction display, the scan was started and a first fixation cross was shown for 10 seconds. Subsequently, a face image was always shown for 2.5 seconds, followed by a jittered fixation cross (0.5 to 6 seconds; average 2 seconds). Children could provide their answer regarding the presence or absence of a red dot while seeing the face image or during the subsequent fixation cross (see Fig. 1b).

### MRI data acquisition

Neuroimaging data were acquired using a GE MR750 MRI scanner with a 32-channel head coil. A high-resolution T1-weighted structural image was obtained for each participant (TR/TE/TI = 2,500/3/1,100 ms; flip angle = 8°; 192 slices; slice thickness = 1.10 mm; FOV = 220 mm). Functional data during the tasks were obtained using an echo-planar imaging (EPI) pulse sequence (TR/TE = 2,400/30 ms; flip angle = 85°; voxel size = 2.41 × 1.8 × 3.2 mm; 38 slices; FOV = 235 mm).

### MRI data preprocessing and analysis

Functional images were analyzed using SPM8 (Department of Neuroscience, Wellcome Trust Center for Neuroimaging, London, UK; http://www.fil.ion.ucl.ac.uk/spm/software/spm8/), running on MATLAB (The MathWorks, Inc., Natick, MA, USA). Images were visually inspected for potential signal loss due to magnetic field inhomogeneity. After slice timing using the middle slice as a reference to correct for acquisition time differences, images were realigned using a least squares approach and a six-parameter rigid-body registration to correct for head motion. We subsequently performed coregistration to individual structural images using a rigid body transformation in three dimensions, and normalization to the Montreal National Institute (MNI) space using a standard T1-weighted average scan provided by SPM8 (ICBM152; dimensions: 91 × 109 × 91; voxel size: 2 mm^3^; presmoothed to 8 mm). Finally, spatial smoothing with a 7-mm full width, half-maximum (FWHM) isotropic Gaussian kernel was performed. As this study involved children, two additional preprocessing steps to detect and repair signal artifacts due to low frequency drifts in mean global signal and rapid scan-to-scan head motion were introduced after slice timing and realignment and before coregistration using ArtRepair software (http://cibsr.stanford.edu/tools/human-brain-project/artrepair-software.html; Mazaika, Hoeft, Glover, & Reiss, 2009), developed at CIBSR and available for public download. The despiking step automatically detects and removes spikes and slow variations using clipping and a digital high-pass filter, thereby reducing the “ringing” side effects that may occur when standard high-pass filters encounter large spikes in the data. The rapid scan-to-scan motion repair step automatically detects spike artifacts at times of abrupt motion between two successive scans and replaces them by interpolation. To ensure good quality of scans after rapid scan-to-scan motion repair, fMRI data were only retained if the percentage of interpolated scans per task/block was below 20% (Neely, Walter, Black, & Reiss, 2012; Vrtička, Black, Neely, Shelly, & Reiss, 2013a; Vrtička, Neely, Shelly, Black, & Reiss, 2013b). In order for these two additional preprocessing steps to operate at best performance, another single-subject smoothing step with FWMH = 4 mm was introduced before artifact repair, which, together with the smoothing of normalized images with FWMH = 7 mm, is about equivalent to one FWMH = 8 mm group smoothing.

For both the social feedback processing and the face-morph tasks, preprocessed scans were then analyzed on the subject level using the general linear model (Friston et al., 1995) implemented in SPM8 in an event-related design and high-pass filtered at 1/128 s (0.008 Hz). To do so, separate regressors for each event type were convolved with a canonical hemodynamic response function. For the social feedback processing task, six zero-duration events were modeled, including the dot display on correct and incorrect trials, and the four critical feedback conditions (SFW, SFL, AFW, AFL; Vrtička et al., 2008; Vrtička et al., 2014). For the face-morph task, six zero-duration events were modeled, comprising the six different morph conditions. For both tasks, the six movement parameters from realignment corrections were entered as additional covariates of no interest to account for residual movement artifacts after realignment.

A second-stage random-effect analysis was then performed using a flexible factorial design also comprising the factor subject. For the social feedback processing task, the main effects contrasts of emotional facial expression and written performance feedback, as well as their interaction, were computed as *F* tests. For the face-morph task, the main effects contrast of self–other degree was also computed as an *F* test. *F* tests were derived across the whole brain and thresholded at *p* < .05 false-discovery-rate (FDR) corrected at the voxel level.

### Associations with attachment and participant age

Raw activation (beta) values from any significant clusters (regions of interest; ROIs) found with the flexible factorial whole-brain analysis were subsequently extracted and tested for associations with attachment avoidance and anxiety. For the social feedback processing task, although we attempted to control for task performance (% “Won” trials) by automatically adjusting task difficulty online, we conducted analyses including task performance as an additional covariate of no interest. We found no task performance effects, so task performance was not considered further. We included age as an exploratory covariate of interest and controlled for sex in all analyses. Taken together, the overall statistical models comprised of sex as a fixed factor and (centered) age, avoidance and anxiety scores, and performance as covariates in repeated-measures analysis of variance (ANOVA) models with the factors emotional facial expression (2; smiling, angry) and written performance feedback (2; Won, Lost) for the social feedback processing, and the factor self–other degree (6; 0%, 20%, 40%, 60%, 80, 100%) for the face-morph task. Given separate models were calculated per ROI, FDR correction for multiple comparisons was carried out for the number of ROIs per contrast.

## Results

### Attachment questionnaire

Attachment orientations were evaluated for 23 participants (all participants for whom at least one fMRI task was available). Please refer to Table 1 for attachment avoidance and anxiety descriptives and reliability statistics (Cronbach’s alpha). Attachment scores were positively correlated (Spearman’s *r* = .690, *p* < .001).

**Table 1:** Summary of attachment questionnaire results

	Attachment avoidance	Attachment anxiety
*M*	2.38	1.97
*SD*	1.06	0.78
Min	1.00	1.00
Max	4.36	3.89
Cronbach’s alpha	.943	.871

*Note.* Results for the two attachment dimensions avoidance and anxiety. Provided are the mean (*M*), standard deviation (*SD*), minimum (Min), maximum (Max), and reliability on a scale from 1 to 7. These measures were derived from raw scores

For further analyses using fMRI data, avoidance and anxiety scores were logarithmically transformed due to positive skew, particularly of the anxiety dimension. There were no extreme outliers (three standard deviations below or above the sample mean) after transformation.

### Social feedback processing

#### Behavioral data

The average behavioral performance (% “Won” trials) during the social feedback processing task was 54.91% (*SD* = 2.41%), ranging from 50.78% to 57.81%.

#### Brain activity

**Fig. 2:**
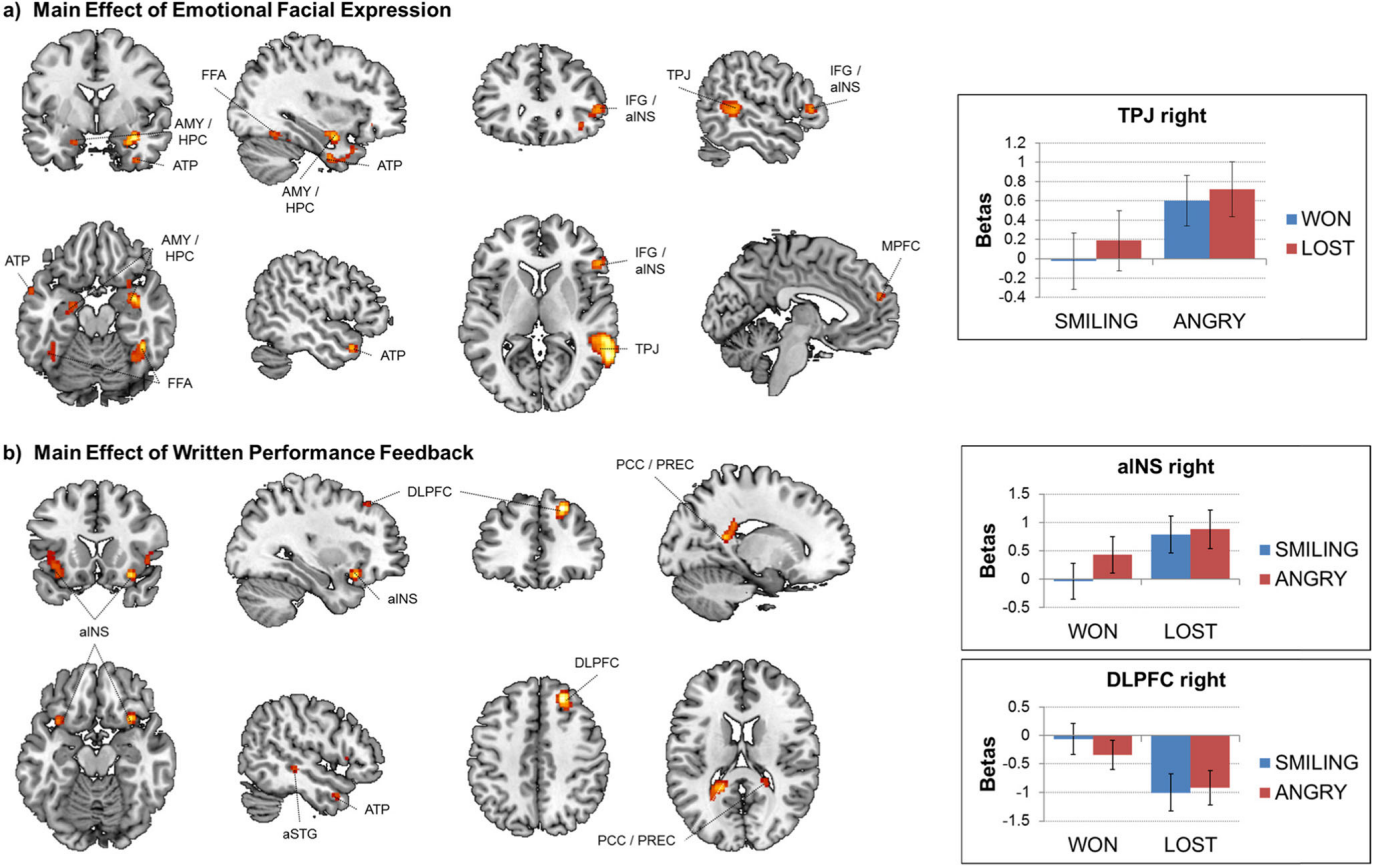
Summary of significant effects observed for the social feedback processing task. **a** Main effect of emotional facial expression showing significant activations in bilateral amygdala (AMY)/hippocampus (HPC), left anterior temporal pole (ATP–two individual clusters), bilateral fusiform face area (FFA), right inferior frontal gyrus (IFG)/anterior insula (aINS), right temporoparietal junction (TPJ), and medial prefrontal cortex (MPFC). Post hoc examination showed that in all of the regions, the direction of the main effect of emotional facial expression was Smiling > Angry, as illustrated on the right by the example plot with extracted raw activation (beta) values from the right TPJ. **b** Main effect of written performance feedback showing significant activations in bilateral aINS, right dorsolateral prefrontal cortex (DLPFC), right ATP, right anterior superior temporal gyrus (aSTG), and bilateral posterior cingulate cortex (PCC)/precuneus (PREC). While in the aINS, aSTG, and ATP, the direction of the main effect of written performance feedback was Won < Lost, the direction was Won > Lost in the DLPFC and PCC/PREC, as illustrated on the right by the example plots with extracted raw activation (beta) values from the right aINS (top) and DLPFC (bottom). Brain activations are shown overlaid on a template single-subject T1 image in MNI space with a statistical threshold of *p* < .05 FDR corrected at the voxel level. Error bars represent 1 *SEM.* (Color figure online)

The flexible factorial design revealed a significant main effect of emotional facial expression (regardless of feedback type), always with the direction Angry > Smiling, on activation in a range of brain areas comprising bilateral amygdala/hippocampus, bilateral fusiform face area (FFA), left anterior temporal pole (ATP; two individual clusters), right temporoparietal junction (TPJ), right inferior frontal gyrus (IFG)/anterior insula (aINS), as well as medial prefrontal cortex (MPFC). There also was a significant main effect of written performance feedback (regardless of emotional facial expression), with the contrast Lost > Won eliciting increased activation in bilateral aINS, right anterior superior temporal gyrus (aSTG), and right ATP, and with the direction Won > Lost eliciting increased activation in right dorsolateral prefrontal cortex (DLPFC) and bilateral posterior cingulate cortex (PCC)/precuneus (see Table 2 and Fig. 2). No significant interaction emerged, however, between the emotional facial expression and written performance feedback conditions.

**Table 2:** Summary of significant effects observed for the social feedback processing task

MAIN EFFECT OF EMOTIONAL FACIAL EXPRESSION						
*k* voxel	*p*-value FDR corrected peak	*F*-value peak	*Z*-value peak	*x, y, z*	Region	Post hoc direction test
725	.036	26.83	4.55	62, −52, 8	TPJ right	Angry > Smiling
371	.036	23.01	4.24	34, −2, −18	AMY/HPC right	Angry > Smiling
204	.036	22.39	4.19	42, −46, −18	FFA right	Angry > Smiling
147	.036	21.37	4.1	−20, −6, −24	AMY/HPC left	Angry > Smiling
49	.036	20.75	4.04	−50, 14, −26	ATP left (1)	Angry > Smiling
148	.036	19.48	3.93	54, 30, 6	IFG/aINS right	Angry > Smiling
39	0.038	17.88	3.77	−4, 54, 22	MPFC	Angry > Smiling
99	0.04	17.5	3.73	−38, −44, −20	FFA left	Angry > Smiling
35	0.042	16.66	3.64	−58, 4, −16	ATP left (2)	Angry > Smiling
MAIN EFFECT OF VERBAL FEEDBACK						
*k* voxel	*p*-value FDR corrected peak	*F*-value peak	*Z*-value peak	*x, y, z*	Region	Post hoc direction test
62	.035	26.6	4.53	32, 18, −16	aINS right	Lost > Won
239	.038	21.73	4.13	−30, 26, −10	aINS left	Lost > Won
22	.038	19.41	3.92	50, −30, −6	aSTG right	Lost > Won
45	.038	19.06	3.89	46, 20, −4	aINS right	Lost > Won
20	.038	18.93	3.87	50, 8, −28	ATP right	Lost > Won
286	.035	30.72	4.82	22, 36, 44	DLPFC right	Won > Lost
302	.035	24.4	4.36	−18, −46, 16	PCC/PREC left	Won > Lost
28	.038	18.24	3.81	20, −40, 16	PCC/PREC right	Won > Lost
INTERACTION						
*k* voxel	*p*-value FDR corrected peak	*F*-value peak	*Z*-value peak	*x, y, z*	Region	Post hoc direction test
*ns*						

*Note*. Provided are the number (*k*) of voxels per cluster, the FDR corrected *p* value of the peak activation, the *F* and *Z* values of the peak activation, the coordinates (*x, y, z*) of the peak activation in MNI space, as well as the best possible estimate of the anatomical region the cluster falls into. The significant main effects of emotional facial expression and written performance feedback were tested post hoc for their respective direction, which is also indicated

**Fig. 3:**
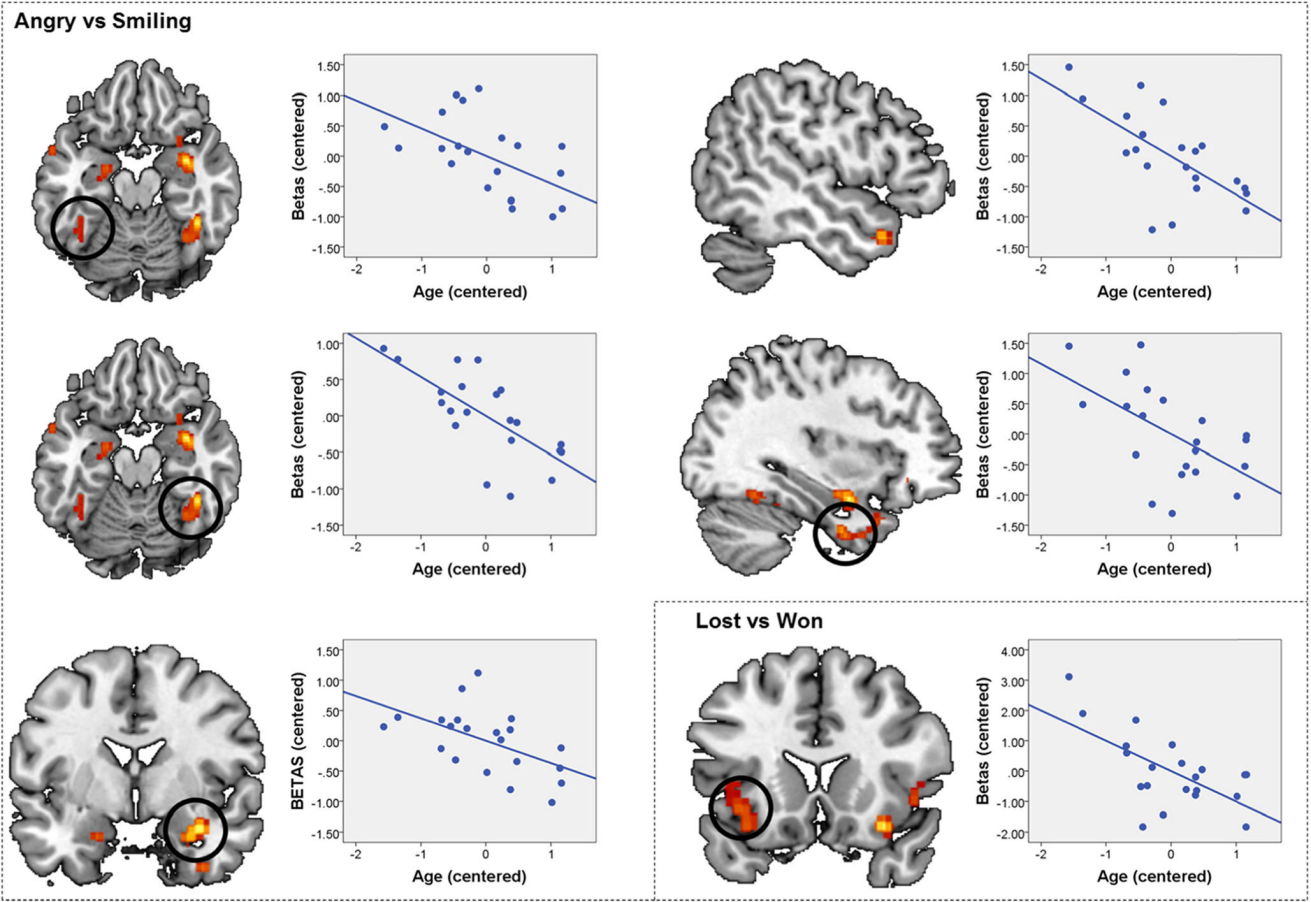
Interactions between brain activity for the main effect of emotional facial expression (angry versus smiling) and verbal feedback (lost versus won) and child age during the social feedback processing task. The difference between extracted raw activation values for the angry versus smile and lost versus won (“betas”) conditions is plotted on the *y*-axis (centered) against age on the *x*-axis (centered). For the main effect of emotional facial expression (angry versus smiling), the pattern is consistent across all five regions of interest described in Table 3 and shows a shift from preferential processing of angry faces in younger children towards smiling faces in older children. Order of regions of interest: top left—left fusiform face area (FFA); middle left—right FFA; bottom left—right amygdala/hippocampus; top right—left anterior temporal pole (ATP; 1); middle right—left ATP (2). For the main effect of verbal feedback, the pattern depicts a shift from preferential processing of lost feedback in younger children towards won feedback in older children in the left anterior insula (aINS). (Color figure online)

The subsequent analyses pertaining to modulation of brain activity within the above ROIs revealed no statistically significant associations with attachment avoidance and anxiety for either contrast. Conversely, we observed a significant positive association between age and brain activity for the Angry > Smiling contrast in the right amygdala/hippocampus, right ATP, and bilateral FFA (see Table 3 and Fig. 3). No significant associations between age and brain activity emerged for the main effect of written performance feedback and the emotional facial expression by written performance feedback interaction. For completeness, effects of child sex and performance are also listed in Table 3, although there were no significant associations. We also ran an additional set of ANOVAs for an analysis where the interaction term between attachment avoidance and anxiety was included, but this did not change the pattern of results.

**Table 3:** Summary of associations between brain activity in regions of interest and participant sex, age, performance, as well as attachment avoidance (AV) and anxiety (AX) during the social feedback processing task

MAIN EFFECT OF EMOTIONAL FACIAL EXPRESSION						
Contrast	Region	AV	AX	Age	% “Won”	Sex
Angry > Smiling	TPJ right	0.521	0.224	0.157	0.667	0.997
Angry > Smiling	AMY/HPC right	0.425	0.305	0.026*	0.805	0.227
Angry > Smiling	FFA right	0.604	0.938	0.001***	0.255	0.182
Angry > Smiling	AMY/HPC left	0.14	0.342	0.036	0.639	0.467
Angry > Smiling	ATP left (1)	0.254	0.07	0.003**	0.206	0.011
Angry > Smiling	IFG/aINS right	0.269	0.036	0.097	0.227	0.851
Angry > Smiling	MPFC	0.492	0.827	0.069	0.739	0.079
Angry > Smiling	FFA left	0.317	0.909	0.021*	0.732	0.415
Angry > Smiling	ATP left (2)	0.936	0.941	0.017*	0.91	0.337
MAIN EFFECT OF WRITTEN PERFORMANCE FEEDBACK						
Contrast	Region	AV	AX	Age	Sex	% “Won”
Lost > Won	aINS right	0.578	0.259	0.116	0.775	0.525
Lost > Won	aINS left	0.903	0.275	0.006*	0.031	0.033
Lost > Won	aSTG right	0.443	0.997	0.384	0.57	0.799
Lost > Won	aINS right	0.884	0.641	0.334	0.315	0.557
Lost > Won	aATP right	0.511	0.379	0.436	0.328	0.831
Won > Lost	DLPFC right	0.176	0.983	0.487	0.694	0.937
Won > Lost	PCC/PREC left	0.767	0.65	0.297	0.724	0.056
Won > Lost	PCC/PREC right	0.418	0.859	0.706	0.886	0.25

*Note*. Direction of the contrast, the respective region of interest, as well as uncorrected *p* values of the respective *F* test from the ANOVA for each variable of interest; *p* values were subsequently checked for significance after FDR correction for multiple comparisons for the number of ROIs, and *p* values that survived this correction are highlighted in bold and emphasized by asterisks (***p < .001, **p < .01, *p < .05 after FDR correction). Abbreviations are explained in the text

### Face-morph task

#### Behavior

Behavioral performance (correct detection of red dots) during the face-morph task was very good—mother–child condition: 96.29% (6.84); stranger-female–child condition: 97.97% (4.57), suggesting that participants were engaged with the task. Average reaction times were 642 (140) ms for the mother–child condition and 615 (105) for the stranger-female–child condition.

#### Brain activity

**Fig. 4:**
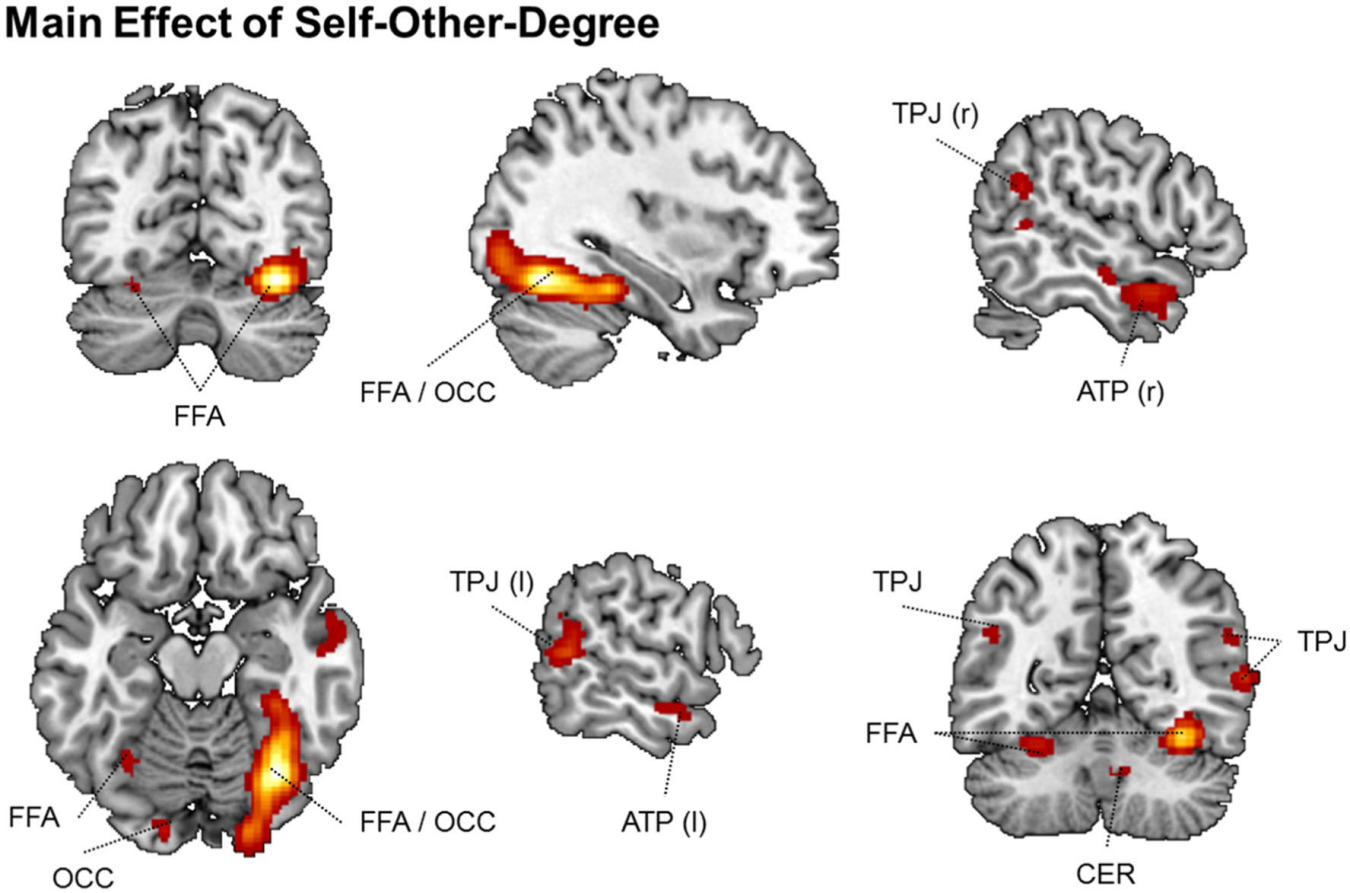
Summary of significant effects observed for the face-morph task, mother–child condition. Main effect of self–other degree showing significant activations in bilateral fusiform face area (FFA)/occipital cortex (OCC), bilateral temporoparietal junction (TPJ), bilateral anterior temporal pole (ATP), and right cerebellum (CER). (Color figure online)

For the mother–child morph condition, the flexible full factorial design revealed a significant main effect of self–other degree in the right FFA extending posterior into the right occipital cortex, bilateral TPJ, left FFA, left occipital cortex, bilateral ATP, and right cerebellum (see Table 4 and Fig. 4).

**Table 4:** Summary of significant effects observed for the face-morph task

MAIN EFFECT OF SELF–OTHER-DEGREE					
*k* voxel	*p*-value FDR corrected peak	*F*-value peak	*Z*-value peak	*x, y, z* peak	Region
2,325	.001	10.7	5.45	34, −68, −16	Fusiform/occipital cortex right
336	.008	6.41	4.01	60, −52, 8	TPJ right
245	.008	6.4	4	−26, −60, −20	FFA left
74	.011	6.18	3.91	−14, −92, −14	OCC left
379	.011	6.14	3.89	54, 6, −22	ATP right
61	.015	5.81	3.75	−58, 4, −26	ATP left
23	.029	5.16	3.45	−48, −56, 26	TPJ left
28	.036	4.96	3.35	10, −60, −32	Cerebellum right

*Note*. Provided are the number (*k*) of voxels per cluster, the FDR-corrected *p* value of the peak activation, the *F* and *Z* values of the peak activation, the coordinates (*x, y, z*) of the peak activation in MNI space, as well as the best possible estimate of the anatomical region the cluster falls into. Abbreviations are explained in the text

**Fig. 5:**
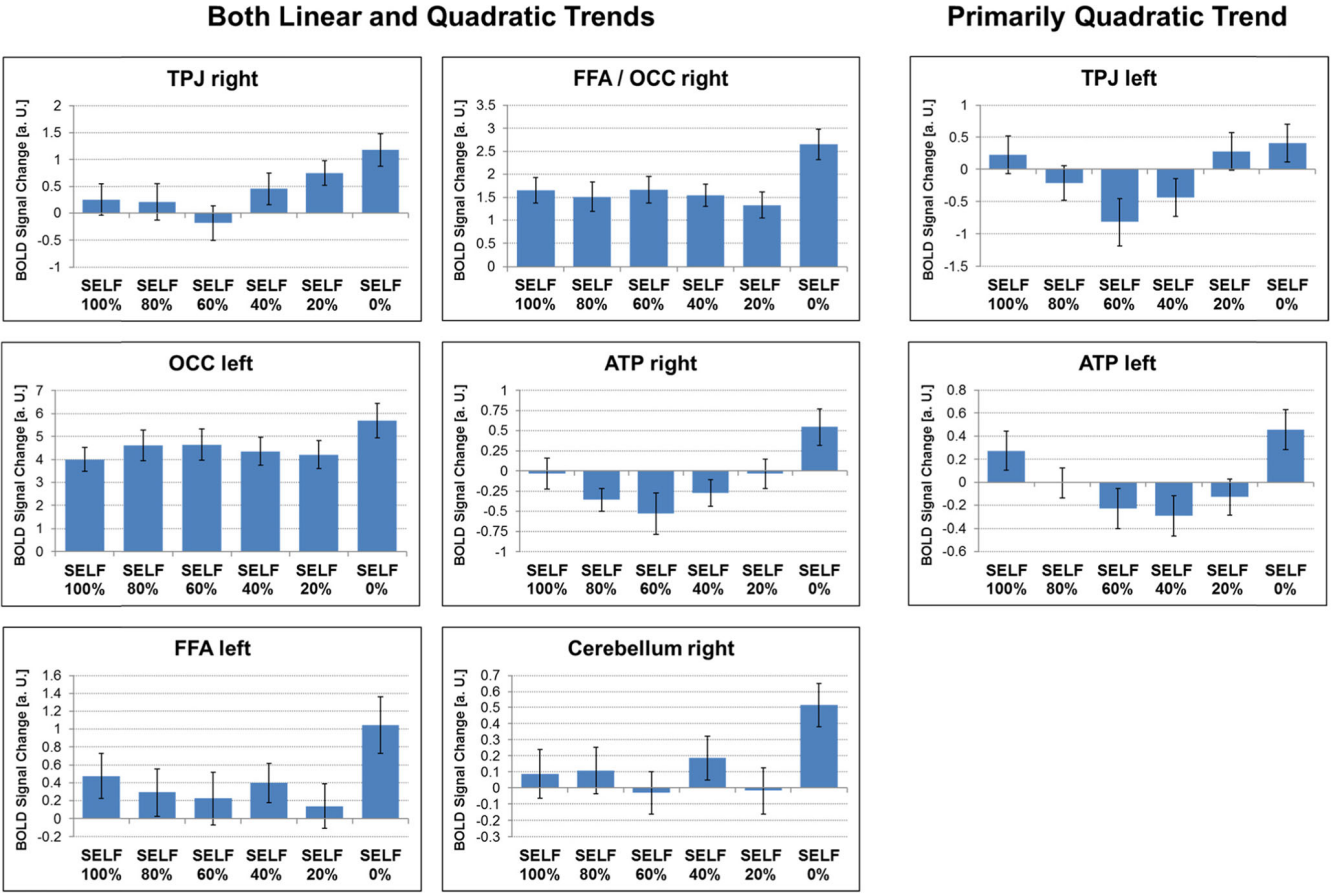
Linear and quadratic trends in the brain activation data of the face-morph task, mother–child condition. Both linear and quadratic trends were found in the right temporoparietal junction (TPJ; top left), right fusiform face area (FFA)/occipital cortex (OCC; top middle), left OCC (middle left), right anterior temporal pole (middle middle), left FFA (bottom left), and right cerebellum (bottom middle). Primarily quadratic trends were present in the left TPJ (top right) and left ATP (middle right). Error bars represent 1 *SEM*; *AU*, arbitrary units. (Color figure online)

Raw activation (beta) values from the above significant clusters were subsequently extracted and tested in SPSS within a repeated-measures ANOVA (with the within-subject factor self–other degree) to further characterize the underlying activation pattern. We tested for the presence of a linear versus quadric pattern of the self–other-degree effect (as assessed from tests of within-subject contrasts). This revealed both linear and quadratic trends for most areas except the left TPJ and ATP, where the linear contribution was not significant and the quadratic contribution was highly significant. In the left TPJ and ATP, the activation pattern resembled a U shape with maximal activation for 100% self and 0% self and minimal activation for the 60% and 40% self conditions, respectively. In turn, in the remaining areas, activity was always highest for the 0% self condition and lower for the remaining conditions (see Table 5 and Fig. 5 as well as Supplementary Table S1 for all post hoc pairwise comparisons).

**Table 5:** Summary of linear and quadratic trends in the brain activation data of the face-morph task, mother–child condition

POST HOC TEST FOR LINEAR AND QUADRATIC TRENDS					
Region/*x, y, z* peak	Order	*F*	*p*	η^2^	Effect
TPJ right	Linear	18.398	<.001	0.467	Both linear and quadratic
60, −52, 8	Quadratic	6.605	.018	0.239	
Fusiform / Occipital right	Linear	10.884	.003	0.341	Both linear and quadratic
34, −68, −16	Quadratic	16.445	.001	0.439	
OCC left	Linear	10.22	.004	0.327	Both linear and quadratic
−14, −92, −14	Quadratic	4.244	.052	0.168	
ATP right	Linear	5.92	.024	0.22	Both linear and quadratic
54, 6, −22	Quadratic	12.894	.002	0.38	
FFA left	Linear	3.59	.072	0.146	Both linear and quadratic
−26, −60, −20	Quadratic	14.899	.001	0.415	
Cerebellum right	Linear	3.271	.085	0.135	Both linear and quadratic
10, −60, −32	Quadratic	9.108	.007	0.303	
TPJ left	Linear	1.789	.195	0.079	Primarily quadratic
−48, −56, 26	Quadratic	11.247	.003	0.349	
ATP left	Linear	0.115	.737	0.005	Primarily quadratic
−58, 4, −26	Quadratic	18.183	<.001	0.464	

*Note*. Region of interest with its peak coordinates (*x, y, z*), the order of the trend, as well as its *F* and *p* values plus η^2^ as an effect-size estimate. Finally, an evaluation based on the strength of linear and quadratic trends is listed under “Effect”

**Fig. 6:**
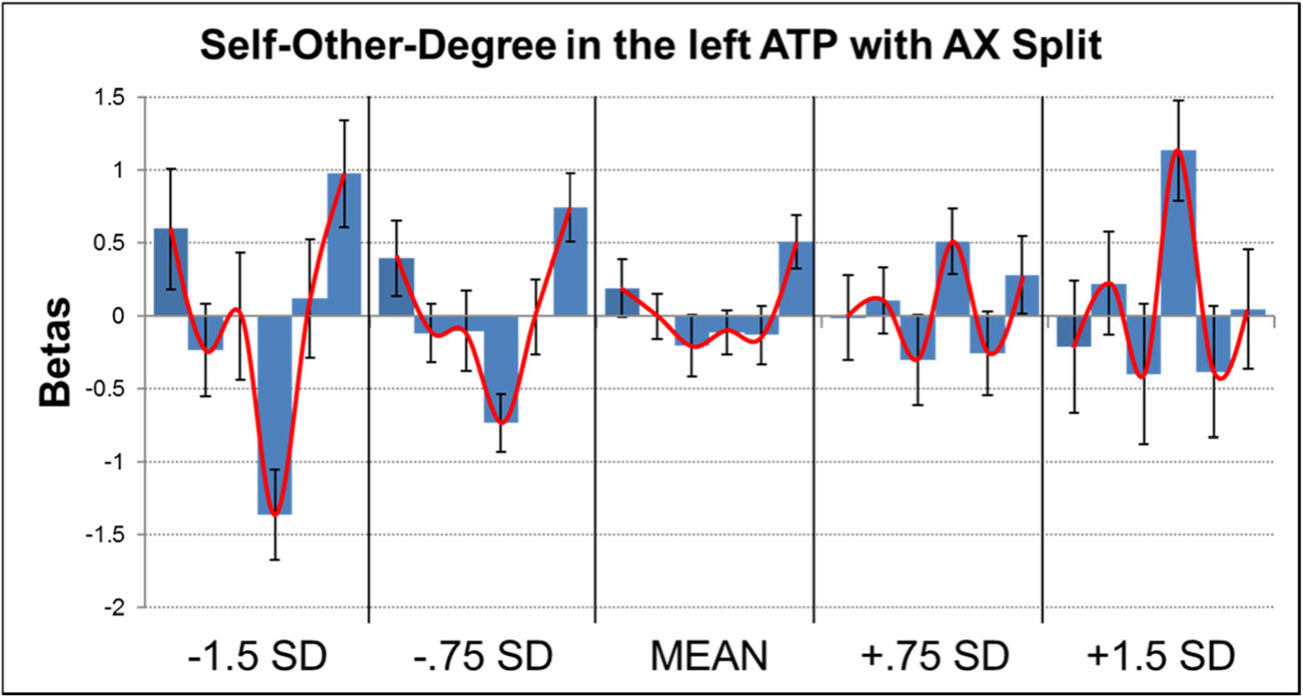
Correlation between activity during the face-morph task, mother–child condition, in the left anterior temporal pole (ATP) and attachment anxiety (AX). Extracted raw activation values (“betas”) are plotted on the *y*-axis for the 100% self, 80% self, 60% self, 40% self, 20% self, and 0% self conditions (from left to right) for AX scores estimated at −1.5 standard deviations (*SD*), −.75 *SD*, the mean, +.75 *SD*, and +1.5 *SD* (on the *x*-axis). Error bars represent 1 *SEM*. The activation pattern is emphasized by a red line connecting the respective mean activations for each self condition. (Color figure online)

In a subsequent step, we again added participant sex, age, as well as attachment avoidance and anxiety to the ANOVAs to test for any relations between the latter measures and brain activity, and once again controlled for multiple comparisons with an FDR correction for the number of ROIs tested. This analysis revealed a selective positive association between brain activity in the left ATP and attachment anxiety (see Table 6). We then tested whether this self–other degree × AX interaction was mainly of a linear or quadratic nature, which showed a primarily quadratic effect (test of within-subjects contrasts; linear: *F* = 0.005, *p* = .924, η^2^ = 0.001; quadratic: *F* = 11.07, *p* = 0.004, η^2^ = 0.394). To visualize this quadratic association between self–other degree and AX, we estimated brain activity within the ANOVA for AX levels at −1.5, −.75, +.75, and +1.5 standard deviations from the mean as well as at the mean, and plotted the results. This revealed that for decreasing AX scores, the quadratic trend more strongly looked like a U shape, whereas for increasing AX scores, the quadratic trend more strongly resembled an inverted U shape (see Fig. 6).

**Table 6:** Summary of associations between brain activity in ROIs during the face-morph task, mother–child condition and attachment avoidance (AV) and anxiety (AX), plus child age and sex

Region	AV	AX	Age	Sex
Fusiform/occipital right	0.738	0.21	0.793	0.656
TPJ right	0.894	0.483	0.919	0.217
FFA left	0.876	0.154	0.581	0.563
OCC left	0.867	0.231	0.748	0.619
ATP right	0.696	0.284	0.853	0.877
ATP left	0.056	0.001**	0.766	0.348
TPJ left	0.777	0.872	0.82	0.558
Cerebellum right	0.471	0.033	0.858	0.066

*Note*. Respective region of interest, as well as uncorrected *p* values of the respective *F* test from the ANOVA for each variable of interest. Uncorrected *p* values were subsequently checked for significance after FDR correction for multiple comparisons for the number of ROIs, and *p v*alues that survived this correction are highlighted in bold and emphasized by asterisks (**p < .01)

We also ran an additional set of ANOVAs for an analysis where the interaction term between attachment avoidance and anxiety was included, but this did not change the results.

For the stranger-female–child morph block, the flexible full factorial design revealed no regions with a statistically significant main effect of self–other degree. Consequently, no follow-up analyses were performed for this block.

## Discussion

Developmental psychology has a rich history of considering attachment-based internal working models (IWMs) and their role in children’s social and emotional development (Bowlby, 1982; Groh et al., 2017). More recently, social neuroscientists have aimed to uncover some of the biological bases of IWMs, but most of this work has focused on adults (Long et al., 2020; Vrtička, 2017). Our study is one of the first to investigate potential relations between attachment, age, and the neural correlates of social-cognitive processing in children. We used two tasks developed in previous neuroimaging studies to assess social feedback processing (Vrtička et al., 2008) and self–other distinction (face-morph task; van Veluw & Chance, 2014). Contrary to previously observed associations in studies of adolescents and adults (Vrtička et al., 2008; Vrtička et al., 2014), we found no associations between attachment and children’s neural response in the social feedback processing task. In the face-morph task, however, anxious attachment was linked with left anterior temporal pole (ATP) activation in the self–mother condition. Specifically, children with less attachment anxiety demonstrated greater left ATP response to either self or mother pictures, which was also the average pattern of ATP activation observed in the sample. Conversely, children with greater attachment anxiety activated the left ATP in response to morphed pictures that combined self and mother. These face-morph task results suggest that in response to self–mother distinction, less versus more attachment anxiety in children may relate to different patterns of activation in the ATP—a region previously associated with mentalizing and emotion processing (Frith & Frith, 2003; Olson, Plotzker, & Ezzyat, 2007). In addition, we observed a shift in activation during the social feedback processing task from (a) negative (i.e., angry) to positive (i.e., smiling) emotional facial expression encoding and (b) negative (i.e., lost) to positive (i.e., won) performance feedback, as a function of an increase in children’s age.

Our findings suggest that in late childhood, neural processing of self–mother recognition may be more relevant than social feedback processing to attachment-based IWMs. Late childhood is a period of sensitivity to parental support (Hostinar, Johnson, & Gunnar, 2015), which could help to explain why we found that children’s anxious attachment was associated with neural processes related to perceiving and representing mother and self. Self–other distinction tasks are thought to tap into self-referential processing (Ma & Han, 2010), which is rooted in attachment-based IWMs (Mikulincer & Shaver, 2007). In accordance with these perspectives, our findings suggest that children’s neural activity during self-referential processing varies as a function of attachment. Conversely, for social feedback processing, neurobiological sensitivity to social evaluation has been found to increase from childhood to adolescence (Stroud et al., 2009). Thus, social feedback processing, which includes a social evaluation component, may become more salient and related to attachment-based IWMs in adolescence. Furthermore, our findings build on previous research that suggest a potential developmental shift from adolescence to adulthood in how attachment-derived IWMs may be connected to the neural substrates of social feedback processing (Vrtička et al., 2008; Vrtička et al., 2014). In our study, the lack of associations with attachment may indicate that social feedback processing, as assessed by our task, did not elicit attachment activation. Based on findings from Vrtička et al. (2014), neural sensitivity to congruent versus incongruent social feedback more globally may be a feature of adolescent IWMs that are still in the process of being shaped through interactions with others. Conversely, more specific activation in reward-related and threat-related systems to congruent combinations of social and objective performance feedback (i.e., social praise and reproach) may characterize IWMs related to attachment system deactivation or hyperactivation in adulthood (Vrtička et al., 2008). Taken together with the current findings, social feedback processing may not emerge as a meaningful correlate of attachment until adolescence, when attachment is more globally related to congruent versus incongruent feedback, and adulthood, when attachment is related to congruent feedback activation in specific brain networks (e.g., reward and aversion systems). An alternative explanation for the absence of associations between attachment and brain activity during the social feedback processing task in late childhood (as compared with previous studies focused on adolescence and adulthood) may be that the task induces different subjective experiences that vary by age. Although we obtained statements on how the social feedback combinations were subjectively perceived in our adult study (e.g., “friends” versus “foes”; see Vrtička et al., 2008), we did not ask children about their subjective experiences in the present study. If the four social feedback combinations were not inducing the same subjective experiences in children as in older participants, or if the task induced less variability in subjective experience in children, then this could have contributed to differences between the current findings and previous findings with adolescents and adults.

In the face-morph task, the specificity of the relation between attachment anxiety and ATP response to the self–mother condition and not the self–stranger condition is interesting, given previous work highlighting overlapping neural representations of self–other as a defining feature of close relationships (Beckes et al., 2013; Cheng et al., 2010). Our findings suggest that increased neural response in the ATP to overlapping representations of self and mother may be characteristic of anxious attachment-based IWMs in late childhood. Conversely, increased neural sensitivity to separate self and mother-representations may characterize more secure attachment-based IWMs. In other words, more anxiously attached children showed increased processing in response to morphed self–mother faces, whereas children with more secure attachments (i.e., lower attachment anxiety) showed increased processing in response to separate self and mother faces. These findings beg the question of whether some children exhibit too much neural sensitivity to overlapping self–mother representations, which would dovetail with some social neuroscience research implicating excessive overlap of self–other representations in anxiety-related responding, such as personal distress in response to others’ pain and needs (Decety & Lamm, 2009). Furthermore, our findings are interesting from the perspective that attachment security is related to the development of an autonomous self (Bowlby, 1969; Mikulincer & Shaver, 2004), which is an important developmental task during the transition from late childhood to early adolescence (Steinberg & Silverberg, 1986). Further research is needed, however, to directly test whether neural activation during self–mother distinction is implicated in children’s developing sense of independence from their parents.

Our findings also shed light on the brain networks involved in social feedback processing and self–mother distinction in late childhood. The brain regions displaying, and experimental conditions that elicited elevated neural activation in our study were different from those found in previous studies with older samples, potentially signaling developmental differences in the brain bases of social feedback processing and self–other recognition in children versus adolescents and adults. In the social feedback processing task, children showed greater neural response to negatively valenced (i.e., loss or angry faces) than positively valenced (i.e., won or smiling faces) material in a number of regions involved in social aversion and negative affect (e.g., amygdala, anterior insula) and mentalizing (e.g., right TPJ, medial PFC). Conversely, Vrtička et al. (2014) found that adolescents showed greater neural response to positively valenced material, particularly in regions important for reward processing, such as the ventral striatum and nucleus accumbens. Furthermore, in another study using the same task with adults (Vrtička et al., 2008), objective feedback (Won) elicited more activation in reward-related regions and overall widespread activation. Interestingly, in our sample of 8-year-old to 12-year-old children, with increasing age, activity shifted from negative (i.e., loss or angry faces) to positive (i.e., won or smiling faces) social-emotional information processing. This pattern more closely resembles those observed in previous studies with adolescents and adults (Vrtička et al., 2008; Vrtička et al., 2014). At the same time, the neural activation patterns in children appeared to be more globally representing incoming social-emotional information by means of facial expressions or performance feedback, but not an integration of these two different channels (i.e., absence of an interaction effect). Our previous data suggest that such integration may only emerge during adolescence by means of a congruency versus incongruency effect, and to become more specific during adulthood, regardless of attachment-derived IWMs. Taken together, these independent cross-sectional studies suggest potential age differences in what children, adolescents, and adults are sensitive to in social feedback processing. As already mentioned above, it may, however, also be the case that the subjective experience induced by the four social feedback conditions varies by age, and/or that there is differential variability in subjective experience as a function of age. Future longitudinal assessments of the neural substrates of social feedback processing should include measurements of subjective experience.

In the self–mother condition of the face-morph task, children showed increased activation to both self and mother faces in the left TPJ and ATP. However, children exhibited increased activation to mother faces compared with self faces in regions previously associated with mentalizing and face processing, including the right temporoparietal junction and bilateral fusiform face area (Frith & Frith, 2003; Vuilleumier, Armony, Driver, & Dolan, 2001). Children may be more prone to differentially engaging these regions when perceiving their mother than when perceiving the self or a stranger, perhaps in the service of spontaneous mental state inference and increased attention. Previous research with adults suggests that cortical midline and right frontoparietal areas exhibit increased activation during self-referential processing (self faces compared with other faces; Uddin, Iacoboni, Lange, & Keenan, 2007). Importantly, however, most studies to date have presented adults with self–other face-morph tasks using unfamiliar strangers. To our knowledge, no fMRI study has examined children’s neural responses to self-distinction tasks using mothers’ faces as stimuli. Our findings suggest that children may recruit the left TPJ and ATP in response to self and mother faces (with a primarily U-shaped activation pattern), but show preferential processing of their mothers’ faces in the right TPJ, occipital, ATP, and FFA (with a primarily linear activation pattern). In our study, such an activation pattern was independent of child’s age, which suggests already present functionality at age 8 and no extended change thereafter up to 13 years. In contrast to social feedback processing, it appears that self-face and mother-face processing may be more stable earlier in development and more relevant to attachment.

There are several study limitations that should be considered. First, our small sample size and overall low levels of attachment anxiety and avoidance may have restricted the ability to detect significant associations between attachment-based IWMs and neural activity. It should also be noted that our analytic approach focused on testing relations of attachment and age with neural activation in regions that showed a significant main effect of condition, or interaction effect between conditions, in the whole-brain analysis across all participants. This analytic choice was mainly due to the nature of the factorial design of the face-morph task, but this approach may have prevented us from detecting associations of attachment and age with neural activation in other regions that did not show a significant main or interaction effect. We therefore cannot rule out alternative explanations for our lack of observed associations between attachment in late childhood and neural activity during social feedback processing. Further studies with greater statistical power and variability in attachment are necessary to confirm our findings or uncover potential associations between IWMs and neural activity related to social cognition and emotion processing. Furthermore, any claims about developmental processes related to age and attachment-derived IWMs from our study and the two independent cross-sectional studies (Vrtička et al., 2008; Vrtička et al., 2014) need further extension and replication using a longitudinal study design, also taking into account subjective experiences (see above). Second, we did not collect data on puberty. Given that pubertal development has been linked with heightened responsivity to social evaluation (Sumter, Bokhorst, Miers, Van Pelt, & Westenberg, 2010; van den Bos, Rooij, Miers, Bokhorst, & Westenberg, 2014), individual differences in pubertal stage may have been particularly relevant for our social feedback processing Task. Third, we used adult faces as stimuli for both tasks, but adult and child emotional faces have been shown to elicit different levels of neural activation in brain regions important for face processing, such as the amygdala (Marusak, Carre, & Thomason, 2013). Peers are a salient source of social information and evaluation in late childhood (Masten, Juvonen, & Spatzier, 2009; Westenberg, Gullone, Bokhorst, Heyne, & King, 2007). Thus, child emotional faces may be a more suitable stimuli for assessing social feedback processing related to children’s experiences with peers. As a related point, measuring attachment to others besides parents, such as peers, would be an interesting future direction for this work. Lastly, although fMRI is a powerful neuroimaging tool, the movement constraints that it places on participants both limits ecological validity and makes it difficult to collect high quality data in younger populations. Functional near-infrared spectroscopy (fNIRS) provides less spatial resolution than fMRI, but it is a promising alternative neuroimaging method for studying cortical underpinnings—and particularly interbrain coherence derived from hyperscanning—of attachment-related processes in children (and their parents; Long et al., 2020; Miller et al., 2019; Nguyen et al., 2020; Vrtička, 2017). Also, fNIRS is suited to investigate other processes relevant in an attachment context that can be measured by hyperscanning, such as trust and cooperation (King-Casas et al., 2005; Reindl, Gerloff, Scharke, & Konrad, 2018). Despite these limitations, this study contributes to a small but growing body of literature aimed at increasing our understanding of the neural processes underlying attachment security in late childhood. These efforts are important for helping to root children’s cognitive schemas about the self, others, and relationships (i.e., attachment-based IWMs) in biology.

### Open practices statement

The data and materials are available on request. However, given the nature of the data and the sample, privacy needs to be considered. The experiment was not preregistered.

## Electronic supplementary material

ESM 1 (DOCX 20 kb)

## References

[CR1] Aron A, Fraley B (1999). Relationship closeness as including other in the self: Cognitive underpinnings and measures. Social Cognition.

[CR2] Beckes L, Coan JA, Hasselmo K (2013). Familiarity promotes blurring of self and other in the neural representation of threat. Social, Cognitive, and Affective Neuroscience.

[CR3] Bowlby J (1969). *Attachment and loss: Vol. 1. Attachment*.

[CR4] Bowlby J (1982). Attachment and loss: Retrospect and prospect. American Journal of Orthopsychiatry.

[CR5] Brenning K, Soenens B, Braet C, Bosmans G (2011). An adaptation of the Experiences in Close Relationships Scale–Revised for use with children and adolescents. Journal of Social and Personal Relationships.

[CR6] Bretherton I, Munholland KA, Cassidy J, Shaver PR (2008). Internal working models in attachment relationships: Elaborating a central construct in attachment theory. *Handbook of attachment: Theory, research, and clinical applications*.

[CR7] Burt KB, Roisman GI (2010). Competence and psychopathology: Cascade effects in the NICHD Study of Early Child Care and Youth Development. Development and Psychopathology.

[CR8] Carlson EA, Hostinar CE, Mliner SB, Gunnar MR (2014). The emergence of attachment following early social deprivation. Development and Psychopathology.

[CR9] Cheng Y, Chen C, Lin C-P, Chou K-H, Decety J (2010). Love hurts: An fMRI study. NeuroImage.

[CR10] Choi EJ, Taylor MJ, Hong S-B, Kim C, Yi S-H (2018). The neural correlates of attachment security in typically developing children. Brain and Cognition.

[CR11] Cooke JE, Stuart-Parrigon KL, Movahed-Abtahi M, Koehn AJ, Kerns KA (2016). Children’s emotion understanding and mother-child attachment: A meta-analysis. Emotion.

[CR12] Debbané M, Badoud D, Sander D, Eliez S, Luyten P, Vrtička P (2017). Brain activity underlying negative self- and other-perception in adolescents: The role of attachment-derived self-representations. Cognitive, Affective, & Behavioral Neuroscience.

[CR13] Decety J, Lamm C, Decety J, Ickes W (2009). Empathy versus personal distress: Recent evidence from social neuroscience. *Social neuroscience: The social neuroscience of empathy*.

[CR14] Fonagy P, Luyten P (2009). A developmental, mentalization-based approach to the understanding and treatment of borderline personality disorder. Development and Psychopathology.

[CR15] Fraley, R. C., Waller, N. G., & Brennan, K. A. (2000). An item-response theory analysis of self-report measures of adult attachment. *Journal of Personality and Social Psychology, 78*, 350–365. 10.1037//0022-3514.78.2.350 10707340

[CR16] Friston KJ, Holmes AP, Poline J-B, Grasby PJ, Williams SCR, Frackowiak RSJ, Turner R (1995). Analysis of fMRI time-series revisited. NeuroImage.

[CR17] Frith U, Frith CD (2003). Development and neurophysiology of mentalizing. Philosophical Transactions of the Royal Society of London B: Biological Sciences.

[CR18] Galinsky AD, Ku G, Wang CS (2005). Perspective-taking and self–other overlap: Fostering social bonds and facilitating social coordination. Group Processes & Intergroup Relations.

[CR19] Groh AM, Fearon RM, van IJzendoorn MH, Bakermans-Kranenburg MJ, Roisman GI (2017). Attachment in the early life course: Meta-analytic evidence for its role in socioeomotional development. Child Development Perspectives.

[CR20] Harris RJ, Young AW, Andrews TJ (2012). Morphing between expressions dissociates continuous from categorical representations of facial expressions. Proceedings of the National Academy of Sciences of the United States of America.

[CR21] Hostinar CE, Johnson AE, Gunnar MR (2015). Parent support is less effective in buffering cortisol stress reactivity for adolescents compared to children. Developmental Science.

[CR22] King-Casas B, Tomlin D, Anen C, Camerer CF, Quartz SR, Montague PR (2005). Getting to know you: Reputation and trust in a two-person economic exchange. Science.

[CR23] Leblanc E, Dégeilh F, Daneault V, Beauchamp MH, Bernier A (2017). Attachment security in infancy: A study of prospective links to brain morphometry in late childhood. Frontiers in Psychology.

[CR24] Letourneau NL, Hart JM, MacMaster FP (2017). Association between nonparenting adult’s attachment patterns and brain structure and function: A systematic review of neuroimaging studies. SAGE Open Nursing.

[CR25] Long, M., Verbeke, W., Ein-Dor, T., & Vrtička, P. (2020). A functional neuro-anatomical model of human attachment (NAMA): Insights from first- and second-person social neuroscience. *Cortex*. doi:10.1016/j.cortex.2020.01.010 10.1016/j.cortex.2020.01.010 32092496

[CR26] Lundqvist, D., Flykt, A., & Öhman, A. (1998). The Karolinksa directed emotional faces [Database of standardized facial images]. Available from Psychology Section, Department of Clinical Neuroscience, Karolinska Hospital, S-171 76 Stockholm, Sweden.

[CR27] Ma Y, Han S (2010). Why we respond faster to the self than to others? An implicit positive association theory of self-advantage during implicit face recognition. Journal of Experimental Psychology: Human Perception and Performance.

[CR28] Marusak HA, Carré JM, Thomason ME (2013). The stimuli drive the response: An fMRI study of youth processing adult or child emotional face stimuli. NeuroImage.

[CR29] Masten CL, Juvonen J, Spatzier A (2009). Relative importance of parents and peers: Differences in academic and social behaviors at three grade levels spanning late childhood and early adolescence. Journal of Early Adolescence.

[CR30] Mazaika, P. K., Hoeft, F., Glover, G., & Reiss, A.L. (2009, June). *Methods and software for fMRI analysis of clinical subjects.* Paper presented at The Organization of Human Brain Mapping, 15th Annual Meeting, San Francisco, CA.

[CR31] Mikulincer M, Shaver PR, Rholes WW, Simpson JA (2004). Security-based self-representations in adulthood: Contents and processes. *Adult attachment: Theory, research, and clinical implications*.

[CR32] Mikulincer M, Shaver PR (2007). *Attachment in adulthood: Structure, dynamics, and change*.

[CR33] Mikulincer M, Orbach I, Iavnieli D (1998). Adult attachment style and affect regulation: Strategic variations in subjective self–other similarity. Journal of Personality and Social Psychology.

[CR34] Miller JG, Vrtička P, Cui X, Shrestha S, Hosseini SMH, Baker JM, Reiss AL (2019). Interbrain synchrony in mother-child dyads during cooperation: An fNIRS hyperscanning study. Neuropsychologia.

[CR35] Natu VS, Barnett MA, Hartley J, Gomez J, Stigliani A, Grill-Spector K (2016). Development of neural sensitivity to face identity correlates with perceptual discriminability. The Journal of Neuroscience.

[CR36] Neely MN, Walter E, Black JM, Reiss AL (2012). Neural correlates of humor detection and appreciation in children. The Journal of Neuroscience.

[CR37] Nguyen, T., Kayhan, E., Schleihauf, H., Matthes, D., Vrtička, P., & Hoehl, S. (2020). The effects of interaction quality on neural synchrony during mother-child problem solving. *Cortex*. doi:10.1016/j.cortex.2019.11.020 10.1016/j.cortex.2019.11.020 31927470

[CR38] Olson IR, Plotzker A, Ezzyat Y (2007). The enigmatic temporal pole: A review of findings on social and emotional processing. Brain.

[CR39] Pallini S, Baiocco R, Schneider BH, Madigan S, Atkinson L (2014). Early child–parent attachment and peer relations: A meta-analysis of recent research. Journal of Family Psychology.

[CR40] Ran G, Zhang Q (2018). The neural correlates of attachment style during emotional processing: An activation likelihood estimation meta-analysis. Attachment & Human Development.

[CR41] Reindl V, Gerloff C, Scharke W, Konrad K (2018). Brain-to-brain synchrony in parent–child dyads and the relationship with emotion regulation revealed by fNIRS-based hyperscanning. NeuroImage.

[CR42] Shaver PR, Mikulincer M (2002). Attachment-related psychodynamics. Attachment and Human Development.

[CR43] Steinberg L, Silverberg SB (1986). The vicissitudes of autonomy in early adolescence. Child Development.

[CR44] Stroud LR, Foster E, Papandonatos GD, Handwerger K, Granger DA, Kivlighan KT, Niaura R (2009). Stress response and the adolescent transition: Performance versus peer rejection stressors. Development and Psychopathology.

[CR45] Sumter SR, Bokhorst CL, Miers AC, Van Pelt J, Westenberg PM (2010). Age and puberty differences in stress responses during a public speaking task: Do adolescents grow more sensitive to social evaluation?. Psychoneuroendocrinology.

[CR46] Swain JE, Kim P, Spicer J, Ho SS, Dayton CJ, Elmadih A, Abel KM (2014). Approaching the biology of human parental attachment: Brain imaging, oxytocin and coordinated assessments of mothers and fathers. Brain Research.

[CR47] Takiguchi S, Fujisawa TX, Mizushima S, Saito DN, Okamoto Y, Shimada K (2015). Ventral striatum dysfunction in children and adolescents with reactive attachment disorder: Functional MRI study. BJPsych Open.

[CR48] Uddin LQ, Davies MS, Scott AA, Zaidel E, Bookheimer SY, Iacoboni M, Dapretto M (2008). Neural basis of self and other representation in autism: An fMRI study of self-face recognition. PLOS ONE.

[CR49] Uddin LQ, Iacoboni M, Lange C, Keenan JP (2007). The self and social cognition: The role of cortical midline structures and mirror neurons. Trends in Cognitive Science.

[CR50] van den Bos E, de Rooij M, Miers AC, Bokhorst CL, Westenberg PM (2014). Adolescents’ increasing stress response to social evaluation: Pubertal effects on cortisol and alpha-amylase during public speaking. Child Development.

[CR51] van Veluw SJ, Chance SA (2014). Differentiating between self and others: An ALE meta-analysis of fMRI studies of self-recognition and theory of mind. Brain Imaging and Behavior.

[CR52] Vrtička P, Ibáñez A, Sedeño L, García AM (2017). The social neuroscience of attachment. *Neuroscience and social science*.

[CR53] Vrtička P, Andersson F, Grandjean D, Sander D, Vuilleumier P (2008). Individual attachment style modulates human amygdala and striatum activation during social appraisal. PLOS ONE.

[CR54] Vrtička P, Black JM, Neely M, Shelly EW, Reiss AL (2013). Humor processing in children: Influence of temperament, age and IQ. Neuropsychologia.

[CR55] Vrtička P, Neely M, Shelly EW, Black JM, Reiss AL (2013). Sex differences during humor appreciation in child-sibling pairs. Social Neuroscience.

[CR56] Vrtička P, Sander D, Anderson B, Badoud D, Eliez S, Debbané M (2014). Social feedback processing from early to late adolescence: Influence of sex, age, and attachment style. Brain and Behavior.

[CR57] Vrtička P, Vuilleumier P (2012). Neuroscience of human social interactions and adult attachment style. Frontiers in Human Neuroscience.

[CR58] Vuilleumier P, Armony JL, Driver J, Dolan RJ (2001). Effects of attention and emotion on face processing in the human brain: An event-related fMRI study. Neuron.

[CR59] Westenberg PM, Gullone E, Bokhorst CL, Heyne DA, King NJ (2007). Social evaluation fear in childhood and adolescence: Normative developmental course and continuity of individual differences. British Journal of Developmental Psychology.

